# Prognosis of different types of acute infection in the first episode of childhood acute leukemia

**DOI:** 10.3389/fped.2025.1589770

**Published:** 2025-05-09

**Authors:** Shasha Li, Shanshan Li, Yi Chen, Shuyuan Jia, Kexin Luan, Feng Cui

**Affiliations:** ^1^ Department of Oncology, The Second Affiliated Hospital of Harbin Medical University, Harbin, China; ^2^ Department of Surgery, The Second Affiliated Hospital of Harbin Medical University, Harbin, China

**Keywords:** prognosis, acute infection, acute pediatric leukemia, acute lymphoblastic leukemia, acute myeloid leukemia, children

## Abstract

**Objective:**

The aim of the present study was to determine the prognosis of different types of acute infection in pediatric leukemia patients.

**Methods:**

A retrospective study was carried out on pediatric leukemia patients with acute infections admitted to the Second Affiliated Hospital of Harbin Medical University between 1 September 2004 and 31 August 2022. Clinical characteristics, diagnostic findings, and prognostic outcomes were extracted from the eligible cases and analyzed.

**Results:**

There were 36 cases of acute myeloid leukemia (AML) and 72 cases of acute lymphoblastic leukemia (ALL) that met the inclusion criteria. There were significant differences in the incidence of pneumonia (47.2% vs. 27.8%, *p* = 0.045) and sepsis (19.4% vs. 2.8%, *p* = 0.006) between the AML and ALL groups. There were 10 cases with a poor prognosis and 26 cases with a favorable prognosis in the AML group. There were no significant differences between the poor prognosis and the favorable prognosis groups except for age (14.2 ± 1.2 years vs. 9.6 ± 4.3 years, *p* = 0.003). There were 14 cases with a poor prognosis and 58 cases with a favorable prognosis in the ALL group. There were no significant differences between the poor prognosis and favorable prognosis groups except for age (13.4 ± 2.7 years vs. 9.2 ± 4.7 years, *p* = 0.002).

**Conclusions:**

There were significantly more incidence of pneumonia and sepsis in children with AML. Younger AML and ALL children with acute infections have more favorable prognoses than older children.

## 1 Introduction

Acute pediatric leukemia is a serious condition in childhood. In 2020, there were 67,008 new reported cases of pediatric leukemia globally, with male patients accounting for 57.85% of all cases (1). The survival rates of pediatric acute lymphoblastic leukemia (ALL) have been reported to be in the range of 83%–94% (2), compared to 65%–70% for pediatric acute myeloid leukemia (AML) (3). Infections may increase morbidity and mortality in patients with acute pediatric leukemia who have a much higher risk of infection, potentially due to their malfunctioning immune system, as well as the burden of their therapies, which could lead to repeated, prolonged, and complicated deterioration of immune cells (4). Infections in acute pediatric leukemia patients, besides causing a higher mortality, also lead to prolonged hospitalizations, disrupt chemotherapy schedules, negatively affect patients’ quality of life, and escalate the demand for extra healthcare resources and costs (5). This multifaceted impact underscores the need for vigilant infection management to optimize treatment outcomes and patient wellbeing.

In this study, we analyzed the prognosis of different types of acute infection in patients with pediatric leukemia and showed the most relevant factors that could affect the prognosis.

## 2 Methods

This retrospective study was conducted in pediatric leukemia patients with acute infections admitted to the Second Affiliated Hospital of Harbin Medical University between 1 September 2004 and 31 August 2022. The inclusion criteria were as follows: (1) patients aged under 18 years; (2) patients diagnosed with acute leukemia according to the established criteria (6, 7); (3) patients experiencing a first episode of leukemia with no prior treatment; (4) patients diagnosed with acute infection based on clinical symptoms and signs, supported by laboratory test results (8); (5) patients with signed informed consent to participate in medical research upon admission. The exclusion criteria were as follows: (1) patients with chronic infectious diseases; (2) patients with other types of cancer; (3) patients with COVID-19 infection, confirmed by nucleic acid amplification tests (NAATs) of nasal swab samples (9, 10); (4) patients with acute conditions needing surgical treatments; and (5) incomplete data.

Clinical characteristics such as age, gender, hospital stay, and different types of acute infections, including upper respiratory tract infection, pneumonia, pleurisy, sepsis, bronchitis, urinary tract infection, oral infection, perianal infection, acute enteritis, herpetic angina, skin infection, and soft tissue infection, were abstracted and analyzed.

Poor prognoses referred to uncontrolled infection or death before discharge from hospital. Favorable prognoses referred to controlled infection or cure of infection before discharge from the hospital.

### 2.1 Statistical analysis

Clinical data were extracted and summarized in an Excel file, then analyzed using SPSS 25.0. The *t*-test was used for continuous data and the chi-square test was used for categorical data. Fisher’s exact test was used when the number in the group was less than five.

The level of significance was set at *p* < 0.05 (two sided).

## 3 Results

There were 36 cases of AML and 72 cases of ALL that met the inclusion criteria. There were no differences between the AML and ALL groups in terms of patient age, gender, hospital stay, upper respiratory tract infection, pleurisy, bronchitis, urinary tract infection, oral infection, perianal infection, acute enteritis, herpetic angina, skin infection, soft tissue infection, or poor prognosis (*p* > 0.05 for all comparisons). There were significant differences in the incidence of pneumonia (47.2% vs. 27.8%, *p* = 0.045) and sepsis (19.4% vs. 2.8%, *p* = 0.006) between the AML and ALL groups (Table 1).

**Table 1:** Clinical characteristics of patients in the AML and ALL groups.

Items	AML + ALL (*n* = 108)	AML (*n* = 36)	ALL (*n* = 72)	*p*
Age (years)	10.3 ± 4.6	10.9 ± 4.2^a^	10.0 ± 4.7^a^	0.337^b^
Male, *n* (%)	74 (68.5%)	24 (66.7%)	50 (69.4%)	0.770^c^
Hospital stay (days)	25.1 ± 16.2	28.6 ± 15.1^a^	23.3 ± 16.4^a^	0.109^b^
Upper respiratory tract infection	50 (46.3%)	12 (33.3%)	38 (52.8%)	0.056^c^
Pneumonia	37 (34.3%)	17 (47.2%)	20 (27.8%)	**0.045** * ^c^
Pleurisy	1 (0.9%)	0 (0)	1 (1.4%)	1^d^
Sepsis	9 (8.3%)	7 (19.4%)	2 (2.8%)	**0.006** * ^d^
Bronchitis	5 (4.6%)	2 (5.6%)	3 (4.2%)	1^d^
Urinary tract infection	2 (1.9%)	1 (2.8%)	1 (1.4%)	1^d^
Oral infection	8 (7.4%)	4 (11.1%)	4 (5.6%)	0.437^d^
Perianal infection	1 (0.9%)	1 (2.8%)	0 (0)	0.333^d^
Acute enteritis	4 (3.7%)	1 (2.8%)	3 (4.2%)	1^d^
Herpetic angina	1 (0.9%)	0 (0)	1 (1.4%)	1^d^
Skin infection	1 (0.9%)	0 (0)	1 (1.4%)	1^d^
Soft tissue infection	1 (0.9%)	1 (2.8%)	0 (0)	0.333^d^
Poor prognosis	24 (22.2%)	10 (27.8%)	14 (19.4%)	0.326^c^

Poor prognosis referred to uncontrolled infection or death. AML, acute myeloid leukemia; ALL, acute lymphocytic leukemia.

Bold values represented *p* < 0.05.

^a^ Mean ± standard deviation.

^b^ *t*-test.

^c^ Chi-square test.

^d^ Fisher’s exact test.

* *p* < 0.05 between AML and ALL.

Patients in the AML or ALL groups were further analyzed based on their prognosis at discharge. There were 10 cases with a poor prognosis and 26 cases with a favorable prognosis in the AML group. There were no significant differences between the poor prognosis and favorable prognosis groups except for age (14.2 ± 1.2 years vs. 9.6 ± 4.3 years, *p* = 0.003) (Table 2).

**Table 2:** Clinical characteristics of patients in the AML group.

Items	Poor prognosis (*n* = 10)	Favorable prognosis (*n* = 26)	*p*
Age (years)	14.2 ± 1.2^a^	9.6 ± 4.3^a^	**0.003** ^b^ *
Male	5 (50%)	19 (73.1%)	0.987^c^
Hospital stay (days)	33.2 ± 12.3^a^	26.8 ± 15.7^a^	0.270^b^
Upper respiratory tract infection	4 (40%)	8 (30.8%)	0.700^d^
Pneumonia	7 (70%)	11 (42.3%)	0.137^c^
Sepsis	4 (40%)	3 (11.5%)	0.076*^d^
Bronchitis	0 (0)	2 (7.7%)	1^d^
Urinary tract infection	1 (10%)	0 (0)	0.278^d^
Oral infection	2 (20%)	2 (7.7%)	0.305^d^
Perianal infection	0 (0)	1 (3.8%)	1^d^
Acute enteritis	1 (10%)	0 (0)	0.278^d^
Soft tissue infection	1 (10%)	0 (0)	0.278^d^

Poor prognosis referred to uncontrolled infection or death before discharged from hospital. Favorable prognosis referred to controlled infection or cure before discharged from hospital. AML, acute myeloid leukemia.

Bold values represented *p* < 0.05.

^a^ Mean ± standard deviation.

^b^ *t*-test.

^c^ Chi-square test.

^d^ Fisher’s exact test.

* *p* < 0.05.

In the same token, there were 14 cases with a poor prognosis and 58 cases with a favorable prognosis in the ALL group. There were no significant differences between the poor prognosis and favorable prognosis groups except for age (13.4 ± 2.7 years vs. 9.2 ± 4.7 years, *p* = 0.002) (Table 3).

**Table 3:** Clinical characteristics of patients in the ALL group.

Items	Poor prognosis (*n* = 14)	Favorable prognosis (*n* = 58)	*p*
Age (years)	13.4 ± 2.7^a^	9.2 ± 4.7^a^	**0.002** ^b^ *
Male	9 (64.3%)	41 (70.7%)	0.641^c^
Hospital stay (days)	26.8 ± 14.9^a^	22.4 ± 16.6^a^	0.380^b^
Upper respiratory tract infection	6 (42.9%)	32 (55.2%)	0.407^c^
Pneumonia	3 (21.4%)	17 (29.3%)	0.744^d^
Pleurisy	0 (0)	1 (1.7%)	1^d^
Sepsis	0 (0)	2 (3.4%)	1^d^
Bronchitis	0 (0)	3 (5.2%)	1^d^
Urinary tract infection	0 (0)	1 (1.7%)	^1^ ^d^
Oral infection	1 (7.1%)	3 (5.2%)	1^d^
Acute enteritis	1 (7.1%)	2 (3.4%)	0.483^d^
Herpetic angina	0 (0)	1 (1.7%)	1^d^
Skin infection	0 (0)	1 (1.7%)	1^d^

ALL, acute lymphocytic leukemia.

Bold values represented *p* < 0.05.

^a^ Mean ± standard deviation.

^b^ *t*-test.

^c^ Chi-square test.

^d^ Fisher’s exact test.

* *p* < 0.05.

The age distribution of prognosis is shown in Table 4. A decreasing trend in favorable prognosis was observed with increasing age in participants in both the ALL and AML groups. There was a significant difference in prognosis between participants aged <12 years and those aged ≥12 years in both cohorts (ALL: *p* = 0.0021; AML: *p* = 0.0004).

**Table 4:** Age distribution of prognosis.

Age (years)	ALL				AML			
	Good (*n*)	Poor (*n*)	Total (*n*)	Good (%)	Good (*n*)	Poor (*n*)	Total (*n*)	Good (%)
1	0	0	0	NA	1	0	1	100
2	4	0	4	100	1	0	1	100
3	6	0	6	100	2	0	2	100
4	4	0	4	100	1	0	1	100
5	3	0	3	100	1	0	1	100
6	6	0	6	100	0	0	0	NA
7	2	1	3	66.7	0	0	0	NA
8	1	1	2	50	2	0	2	100
9	2	0	2	100	2	0	2	100
10	2	0	2	100	4	0	4	100
11	6	0	6	100	3	0	3	100
12	3	2	5	60	2	1	3	66.7
13	5	1	6	83.3	2	2	4	50
14	3	3	6	50	2	2	4	50
15	7	4	11	63.6	2	4	6	33.3
16	3	1	4	75	0	1	1	0
17	1	1	2	50	1	0	1	100
Total	58	14	72	80.6	26	10	36	72.2
<12	36	2			17	0		
≥12	22	12			9	10		
*p* ^a^	**0.0021**				**0.0004**			

Bold values represented *p* < 0.05.

^a^ Fisher’s exact test of prognosis difference between children aged <12 years and those aged ≥12 years.

## 4 Discussion

In another study, ALL was reported as the most common type of pediatric acute leukemia, accounting for approximately 75%–80% of cases (11). In our study, 66.7% of pediatric leukemia patients were diagnosed with ALL. This discrepancy might be due to differences in the inclusion and exclusion criteria, as well as variations related to environmental factors and racial demographics.

The most common acute infection observed among the enrolled acute leukemia patients in our study were upper respiratory tract infection (46.3%), followed by pneumonia (34.3%), sepsis (8.3%), and oral infections (7.4%). Neutropenia emerged as the leading risk factor for infection development (12). In addition, several other factors contribute to increased infection risk, including compromised cellular or humoral immunity, disruption of natural barriers (such as skin and mucous membranes), and the use of medical devices, such as vascular access catheters (13). Often, patients are affected by a combination of these risk factors, which can further elevate their susceptibility to infections and the risk of adverse outcomes.

We found that pneumonia (47.2% vs. 27.8%, *p* = 0.045) and sepsis (19.4% vs. 2.8%, *p* = 0.006) were significantly more common in AML than in ALL cases. This can be explained by the differing immunological deficits associated with each leukemia type. In AML, deficits in neutrophilic granulocytes lead to a higher incidence of bacterial and fungal infections, whereas in ALL, deficits in lymphocytes result in hypogammaglobulinemia, leading to reduced cell-mediated immunity (14).

During treatment for ALL and AML, different agents and schedules might be employed, leading to differences in prognosis (14, 15). Both the disease and its therapies place a heavy burden on the developing immune system. In acute leukemia, normal production of blood cells in the bone marrow is disrupted (16, 17) and leukemia cells crowd out healthy white blood cells, including lymphocytes and granulocytes, which are both essential for immune defense. This results in children experiencing frequent infections and fevers, as the body struggles to mount an effective response against pathogens.

Treatment for acute leukemia, primarily via chemotherapy, further exacerbates immune dysfunction (18–21). Chemotherapy targets rapidly dividing cells, affecting not only cancer cells but also healthy immune cells, leading to suppression of the immune system. This increases children’s susceptibility to infections both during and after treatment. The effects of chemotherapy on the immune system can persist even after treatment completion, as evidenced by persistent abnormalities in immune parameters such as lymphocyte subsets and natural killer cell function. In the context of AML, children often experience multiple episodes of infection during intensive treatment, with sepsis being the most common (15). Infection-related mortality rates are in the range of 5.4%–7.3%. In ALL, the induction and consolidation phases pose significant risks for infections due to severe neutropenia. Infection-related mortality in ALL is generally lower, in the range of 2%–4%; however, infections remain a primary cause of treatment-related mortality. Understanding these risks is crucial for developing effective strategies to manage and prevent infections during leukemia treatment.

It is also interesting to note that a previous study in children and adolescents with ALL showed there were no significant associations between sex, race, age, and the development of acute respiratory infections (22). Another study investigating childhood AML with infections showed that age above 16 years was a factor associated with infection-related mortality (23). In general, independent of acute infections, the survival rate of ALL is highest when children diagnosed at 1–4 years of age, with a decline observed in older age groups. Infants aged under 1 year have the lowest survival rate in both ALL and AML (24). In our study, children aged under 12 years demonstrated a more favorable prognosis in both ALL and AML populations. The underlying pathophysiological mechanisms need further investigation and might be related to deficiencies of key factors in metabolism (25, 26).

The present study has some limitations. Notably, the sample sizes in certain subgroups—particularly when comparing infection type and age—were relatively small. Therefore, the conclusions drawn need to be validated in more robust studies, such as meta-analyses or large-scale studies.

## 5 Conclusion

In this study, by analyzing clinical data collected over an 18-year period at our hospital, we found a significantly higher incidence of pneumonia and sepsis in children diagnosed with AML compared to those with ALL. Younger children with AML or ALL who developed acute infections tended to have a better prognosis than older children.

## Data Availability

The raw data supporting the conclusions of this article will be made available by the authors, without undue reservation.
